# Characterising the Microstructure of an Additively Built Al-Cu-Li Alloy

**DOI:** 10.3390/ma13225188

**Published:** 2020-11-17

**Authors:** Iris Raffeis, Frank Adjei-Kyeremeh, Uwe Vroomen, Silvia Richter, Andreas Bührig-Polaczek

**Affiliations:** 1 Foundry Institute, RWTH Aachen University, Intzestraße 5, 52072 Aachen, Germany; f.kyeremeh@gi.rwth-aachen.de (F.A.-K.); u.vroomen@gi.rwth-aachen.de (U.V.); sekretariat@gi.rwth-aachen.de (A.B.-P.); 2 Central Facility for Electron Microscopy (GFE), RWTH Aachen University, Ahornstraße 55, 52074 Aachen, Germany; richter@gfe.rwth-aachen.de

**Keywords:** Al-Cu-Li, additive manufacturing, LPBF, S-HEXRD, EPMA, LOM, SEM, EBSD

## Abstract

Al-Cu-Li alloys are famous for their high strength, ductility and weight-saving properties, and have for many years been the aerospace alloy of choice. Depending on the alloy composition, this multi-phase system may give rise to several phases, including the major strengthening T_1_ (Al_2_CuLi) phase. Microstructure investigations have extensively been reported for conventionally processed alloys with little focus on their Additive Manufacturing (AM) characterised microstructures. In this work, the Laser Powder Bed Fusion (LPBF) built microstructures of an AA2099 Al-Cu-Li alloy are characterised in the as-built (no preheating) and preheat-treated (320 °C, 500 °C) conditions using various analytical techniques, including Synchrotron High-Energy X-ray Diffraction (S-HEXRD). The observed dislocations in the AM as-built condition with no detected T_1_ precipitates confirm the conventional view of the difficulty of T_1_ to nucleate on dislocations without appropriate heat treatments. Two main phases, T_1_ (Al_2_CuLi) and T_B_ (Al_7.5_Cu_4_Li), were detected using S-HEXRD at both preheat-treated temperatures. Higher volume fraction of T_1_ measured in the 500 °C (75.2 HV_0.1_) sample resulted in a higher microhardness compared to the 320 °C (58.7 HV_0.1_) sample. Higher T_B_ volume fraction measured in the 320 °C sample had a minimal strength effect.

## 1. Introduction

The aerospace industry‘s interest in Al-Li alloys dates back to the 1950′s, and this is attributed to their high strength and weight-saving potential, based on the unique property of the Al-Li system where there is a density reduction of 3% and an increase in the elastic modulus between 5% to 6% with each wt.% of Li added [1]. This alloy group generally possesses remarkable fatigue toughness and cryogenic properties that make them suited for applications in aircrafts [2]. Although there are no commercially known binary Al-Li alloys, a number of alloy additions have led to the development of several high-strength Al-Li alloys [3]. One of such high-strength Al-Li alloys is the precipitation hardenable 2XXX series Al-Cu-Li alloy. With Cu as the principal alloying element, minor alloying element possibilities like Mg, Ag, and Zr or their combinations can lead to a multi-phase system of various strengthening phases such as Al_3_Li (δ/δ’), Al_3_Zr, Al_2_Cu (θ/θ’), Al_2_CuLi (T_1_) or Al_2_CuMg (S’) [4]. The main strengthening phase notable among them is the T_1_ phase, who’s strengthening effect, when maximized, leads to significant improvement in strength and ductility of the alloy [5].

The principal strengthening effect of the semi-coherent T_1_ phase is reportedly due to the resulting interfacial energies between the boundaries of the T_1_ plates and the matrix, which generates high lattice strains [6]. In an earlier work done by the authors [7], they asserted that the T_1_ plate which lies on the {111} matrix planes, through their broad coherent face, applies more pressure on incoming dislocations rather than with their thin incoherent face. They postulated that any dislocation impingement would rather take place through the path of least resistance, in this case, the thin incoherent face, making the thickness of the plate vital to the strength of the alloy. The authors [8] observed that increasing T_1_ plate thickness led to decreasing yield strength, but a constant thickness of high-volume fraction T_1_ rather led to an increase in yield strength in an AA2198 alloy. Depending on the alloy composition and the thermomechanical treatment employed, other phases such as T_B_ (Al._7.5_Cu_4_Li), T_2_ (Al_6_CuLi_3_) or R (Al_5_CuLi_3_) phase may form [3].

T_1_ precipitate found in conventionally processed Al-Cu-Li alloys has a platelet morphology, hexagonal crystal structure, with a p6/mmm symmetry which occurs on the {111}_Al_ crystallographic planes [9]. According to Reference [10], T_1_ in conventionally processed Al-Cu-Li alloys require prior deformation by cold working, which induces dislocations that act as nucleation sites for its precipitation. The authors further concluded that increasing the dislocation density through various degrees of cold working prior to appropriate artificial ageing increases the volume fraction of finely distributed T_1_, which consequently improves the strength and ductility of the alloy. Deformation by cold working ahead of artificial ageing is therefore a prerequisite to achieving peak mechanical properties in conventionally processed Al-Cu-Li alloys [5].

In work done in Reference [8] on an AA2198 Al-Cu-Li alloy, the authors reported that through a combination of various degrees of pre-deformation and artificial ageing, mean thickness and diameter of 1.3 and 40 nm at 155 °C ageing temperature respectively, increased to 2 and 55 nm at 190 °C. They also reported a volume fraction of between 0.3% and 3.5% with a conclusion that phases such as ϴ’ and S were only significantly found in non-pre-deformed samples. From investigations by the authors of Reference [11], an average T_1_ diameter of between 49 and 51 nm and an average thickness of between 1.1 and 1.3 nm were reported after various degrees of pre-deformation and different ageing conditions, suggesting possible formation of Guiner-Preston (GP) Zones (GP I/GP II) or ϴ’ phase. From thermal stability investigations on an AA 2099 Al-Cu-Li alloy in Reference [12], the authors concluded that T_1_ was thermally stable and was responsible for the strength of the alloy after over-ageing between 200 and 305 °C. Up to about 200 nm of T_1_ diameter was observed in their work. Using an annular dark-field scanning transmission electron microscopy (ADF-STEM), the authors also confirmed the presence of other phases such as the S, ϴ’ and δ’ after a T83 heat treatment. In studies conducted in Reference [13] using Small-Angle X-ray Scattering (SAXS) and High-Angle Annular Dark Field Scanning Transmission Electron Microscopy (HAADF-STEM) imaging investigations on the structure of T_1_, found T_1_ to be very thin in thickness, largely less than 1 nm. They also found streaks in which they attributed to ϴ’ precipitates. A 5–10 nm diameter of T_1_ occurring on the {111} planes was reported by the authors of Reference [14], who investigated microstructural evolution of an AA2198 Al-Cu-Li alloy using Atom Probe Tomography (APT). Phases such as S and ϴ’ were equally observed at various ageing temperatures. Using APT and Transmission Electron Microscopy (TEM) to characterize a T8 processed AA 2195 Al-Li alloy, the authors [15] concluded that the composition of T_1_ was non-stoichiometric and that it deviated from the stoichiometry (Al_2_CuLi) of the bulk equilibrium T_1_ phase. The authors further concluded that the T_1_/matrix interface is one of Al-Cu layer rather than an Al-Li layer.

The structure and morphology of T_B_ phase was also characterised by the authors of References [16,17] as rod-like with lattice parameters a = 0.583 nm and a [100]_TB_ || [ll0]_AI_ and [001]_TB_ || [001]_AI_ orientation with the Al matrix in an Al-Cu-Li alloy.

These investigations and many more have been carried out using conventional manufacturing methods through a combination of thermomechanical processes with various analytical techniques. A huge research gap however exists as far as Additive Manufacturing (AM) processing of Al-Li alloys. The qualification of Al-Cu-Li alloys for AM applications will therefore require a comprehensive characterization of their AM-built microstructures, which will bring to the fore the requisite knowledge and understanding for maximizing their strength properties. Due to the enormous AM process advantages compared to conventional manufacturing methods, such as the wide design and fabrication freedom as well as tool-free manufacturing, which helps to eliminate many production steps, among others [18], it is expedient to focus research attention not only on processability, but also microstructure characterisation and their correlation to mechanical properties of the AM-built Al-Cu-Li alloys. The generation of finer microstructures due to the high cooling rates, which is characteristic to AM processes like Laser Powder Bed Fusion (LPBF), usually leads to comparable or even better mechanical properties than conventional methods, consistent with the Hall-Petch relationship [19]. Maximizing the strength potential of the major strength contributing T_1_ phase as well as other strengthening phases in AM-processed Al-Cu-Li alloys, will present AM as a process route of choice for high-strength and lightweight applications.

In our earlier work [20], we first successfully reported the LPBF processability of the AA2099 Al-Cu-Li alloy with relative density of 98.8% and a micro-Vickers hardness (HV) of 72 HV_0.1_ using a preheat treatment temperature of 520 °C. The alloy’s recorded microhardness was suspected to be due to the presence of the T_1_ phase, although no phase characterisation was performed. In a recent work carried out by the authors of Reference [21] on LPBF processability, microstructure and mechanical properties of an AA 2195 Al-Li alloy, the authors focused on as-built (no preheating) and T6 post-heat-treated conditions. They also attributed mechanical properties to T_1_ precipitates.

In this work, however, focus is given to the characterisation of the microstructure and microstructure phases using a combination of several analytical instruments as well as the quantification of phase volume fractions. The correlation with the microhardness of as-built and preheat-treated LPBF samples is also reported. The influence of preheat treatment as opposed to any form of post-heat treatment on the phase precipitation of the precipitation hardenable AA 2099 alloy is preferred in this investigation.

## 2. Materials and Methods

The alloy considered under this study, AlCu2.7Li1.8Mg0.3 (AA2099) Al-Cu-Li alloy, was gas-atomized by Nanonval GmbH (Berlin, Germany) under argon (Ar) inert atmosphere. The nominal composition (wt.%) indicating a Cu/Li ratio of 1.3 is highlighted in Table 1 below.

**Figure 1:**
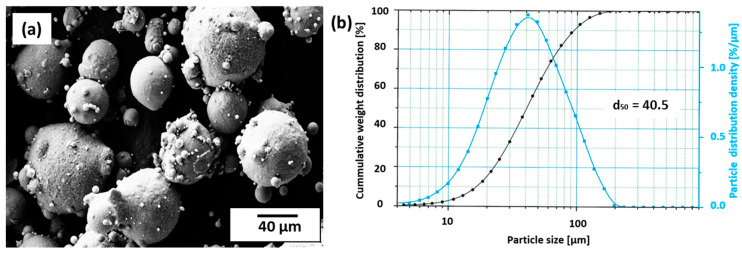
(**a**) Morphology of the gas-atomized particles with few satellites. (**b**) Particle size distribution (PSD) graph of the AlCu2.7Li1.8Mg0.3 alloy recording a d_50_ of 40.5 µm.

Particle size distribution (PSD), as illustrated in Figure 1, is a graph showing the superimposition of the cumulative particle distribution on the density distribution of the particles. The particles recorded high average aspect ratio and symmetry of 0.84 and 0.92 respectively, as well as a median particle diameter (d_50_) of 40.5 µm. They were generally spherical in morphology with satellites, recording an average sphericity of 0.8.

Nominal composition of the powder was determined with the Spectro ARCOS Inductively Coupled Plasma Optical Emission Spectroscopy (ICP-OES) (SPECTRO Analytical Instruments GmbH, Kleve, Germany). Using Retsch Technology GmbH‘s CAMSIZER X2 (Haan, Germany), particle size analysis was performed. Microstructure analyses were carried out using the Zeiss Light Optical Microscope (LOM) (Carl Zeiss Microscopy GmbH, Jena, Germany) and the Zeiss Scanning Electron Microscope (SEM); Field Emission Gun (FEG) (Carl Zeiss Microscopy GmbH) with Oxford Instrument Inca X-sight Energy Dispersive Spectroscopy (EDS)/Electron Backscatter Diffraction (EBSD) detectors (Oxford Instruments GmbH, Wiesbaden, Germany). Etching was performed with 0.5% hydrofluoric (HF) acid for the AM-built samples for 15 s. Relative densities of LPBF-built samples were determined with FIJI IMAGEJ open source software (Version 2). Due to the low atomic number of lithium and hence its low characteristic energy radiation, making it not detectable by Energy Dispersive Spectroscopy (EDS), Synchrotron High-Energy X-ray Diffraction (S-HEXRD) at the Deutsches Elektronen-Synchrotron (DESY) (Hamburg, Germany) was used for phase characterisation. Beamline P02.1 (wavelength of 0.20682Å) with a PerkinElmer XRD1621 fast area detector was used for the experiment. The European Synchrotron Radiation Facility (ESRF), (Grenoble, France) Fit2D software (Version 18 beta), was utilised for post-processing of the results from two (2)-dimensional patterns (2D) to 1-dimensional diffraction peaks (1D) with a 2ϴ ring pattern range of 2.85 Å to 11.35 Å. MAUD (Material Analysis Using Diffraction) open source software (Version 2.94), was used for refinement and fitting as well as phase characterisation and quantification. Bright field mode (BF) of the FEI Tecnai F20 JEOL JEM 2000 FX II Transmission Electron Microscope (TEM) was utilised to detect dislocation presence. The JEOL JXA-8530F field-emission electron microprobe analyser (EPMA) (JEOL Limited, Japan) was also used for local elemental analysis of segregations on the grain boundary, especially of Cu for both the powder and AM solidly built sample.

A Computer-Aided Design (CAD) test sample model of 5 × 5 × 10 mm^3^ (Length × Breadth × Height) dimension designed with the Materialise Magics 24 CAD software (Gilching, Germany) was printed using the Aconity MINI (IPG Photonics YLR 400, F-Theta lens (f = 420 mm, λ = 1030–1080 nm) Laser Powder Bed Fusion (LPBF) equipment (Herzogenrath, Germany) with a beam diameter of 70 µm. This was done under an inert Argon (Ar) gas atmosphere. The test samples were built with a bidirectional XY scanning strategy which turned about at 90 °C on each layer. Seven (7) test samples were printed both in the as-built and preheat-treated conditions to obtain the optimum process parameters, based on which, the densest test samples were chosen for microstructural analytics. The preheat treatment temperatures chosen for the purposes of these investigations were 320 and 500 °C based on the Scheil-Gulliver ThermoCalc (Calculation of Phase Diagrams) CALPHAD-based phase simulation (TCAL6 database). The process parameters which yielded the densest test samples both in the as-built and preheat-treated conditions are highlighted in Table 2 below.

Microhardness measurements were performed with the Buehler Microhardness testing equipment (Esslingen, Germany) using a load of 100 g (0.1 N). The measurements were taken on all the seven printed samples, on both the as-built and preheat-treated samples. The measurement on each sample was determined by averaging nine sets of microhardness measurements: three each at the bottom, middle and top.

## 3. Results

### 3.1. Powder Characterisation

**Figure 2:**
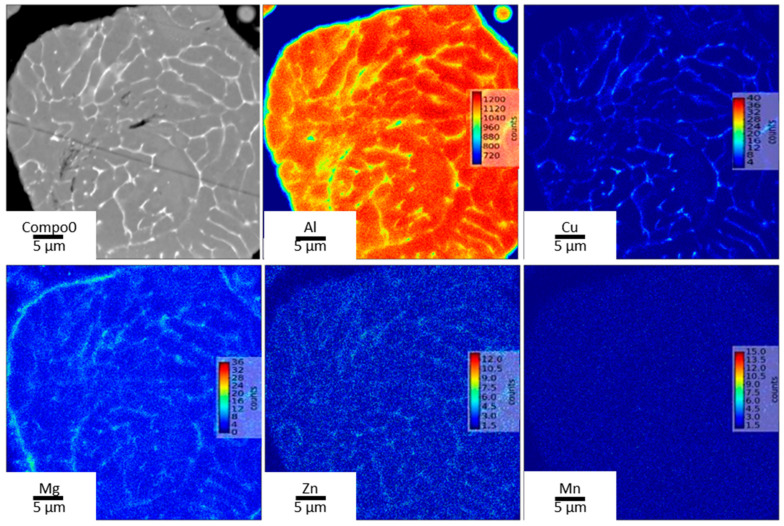
Electron Probe Micro Analyser (EPMA) maps of a cross-sectioned powder particle showing inter-dendritic phase with high Cu enrichments and significant concentrations of Zn and Mg.

In the cross-section of the gas-atomized powder shown by the EPMA maps in Figure 2, the microstructures show inter-dendritic phases arising from solidification of the melt droplets during the gas atomization. This was similarly reported in Reference [22] in a gas-atomized Al-Cu alloy with 4 wt.% Cu, and the authors attributed it to partitioning of solute between the liquid and solid phase during solidification. From the EPMA maps, these inter-dendritic phases were found to possess high Cu with significant amounts of Zn and Mg segregations. Mn, however, is seen to be have been homogenously dissolved in the matrix. Owing to the small compositional amount of Li available in the alloy, a local EPMA map of Li could not be generated.

### 3.2. Microstructure of As-Built Sample

**Figure 3:**
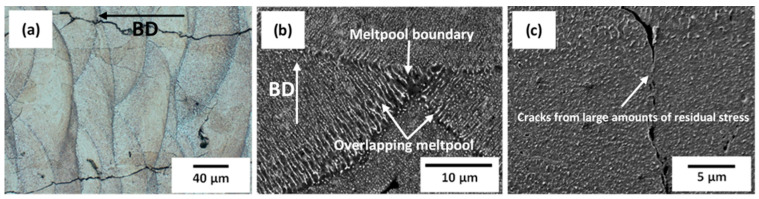
As-built LPBF microstructures: (**a**) Observed under Light Optical Microscope (LOM), (**b**) observed under Scanning Electron Microscope (SEM), showing melt-pool boundary and melt-pool boundary overlaps and (**c**) observed residual stress-induced cracks in the as-built condition.

The microstructure of as-built samples shown in the LOM micrograph in Figure 3a, is consistent with as-built microstructures with overlapping melt-pool boundary and with columnar orientation along the building direction. The preferential growth of columnar grains in additive manufacturing during solidification is driven by the steep temperature gradient (G) and the extremely high cooling rates (T) [23]. Columnar grains introduce anisotropic properties in AM-built parts and may be detrimental for multidimensional stress applications, however with inoculation with potent nucleants or the adjustment of process parameters to control temperature gradient and melt velocity, equiaxed grains can be promoted to yield isotropic properties in AM-built parts [24,25]. The steep temperature gradient usually results in the build-up of large amounts of residual stresses in the as-built condition, which may lead to fracture when the local tensile strength is exceeded [26]. As shown in Figure 3a, the residual stress-induced cracks are clearly seen in the as-built sample. Several options are available for minimizing residual stresses in LPBF such as the adoption of in-situ heat treatment, post-fabrication stress-relief mechanisms (post-heat treatment) or the lowering of the high-temperature gradient through variation of scanning strategy between the scan tracks [27]. Post-heat-treated as-built samples at various temperatures proved to be detrimental to the mechanical properties (hardness), as opposed to in-situ heat treatment [20].

**Figure 4:**
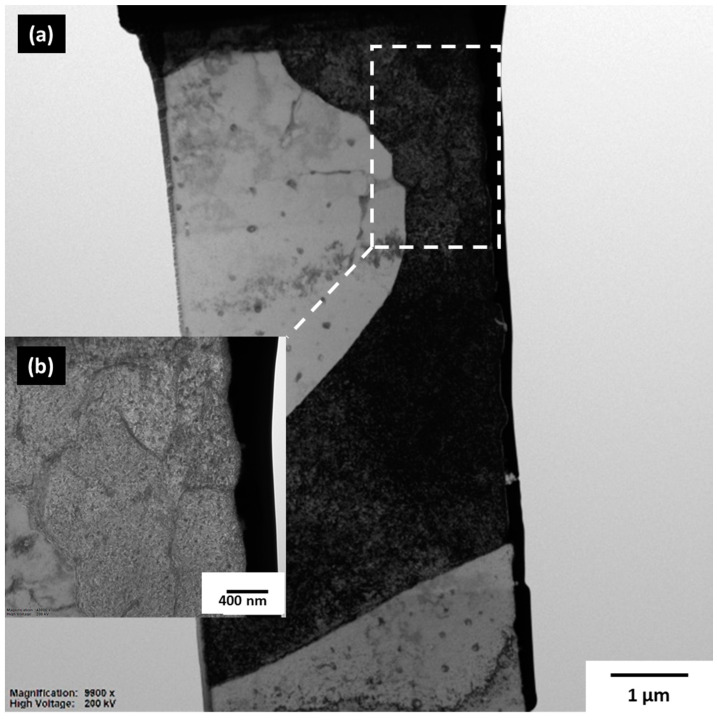
(**a**) Bright Field (BF)-Transmission Electron Microscope (TEM) micrograph showing dislocations in the as-built sample taken at 200 KV. (**b**). Insert: BF-TEM image taken at high magnification (43,000, 200 kV).

The presence of dislocations in as-built LPBF samples have been reported several times by various authors [19,28,29]. According to the authors of Reference [19], who found many dislocation networks in as-built duplex stainless-steel, the high dislocation density in as-built LPBF samples can be compared to prior deformation by cold working, which gives rise to many dislocation networks, as seen in typical conventional manufacturing methods, including the alloy under investigation. As mentioned earlier, conventional processing of Al-Cu-Li alloys such as the AA 2099 alloy requires various degrees of prior deformation by cold working, with appropriate artificial aging treatment to be able to precipitate a high-volume fraction of the main strengthening T_1_ precipitates. In Figure 3b,c, the SEM micrographs show no presence of T_1_ plate formation even at a highly observed magnification of 10,000. In the BF mode, using Transmission Electron Microscope (TEM) micrographs, dislocations in the as-built sample were found, as shown in Figure 4a,b.

It stands to reason that, although dislocations induced by the large amounts of residual stress in the as-built condition, which could have acted as potent T_1_ nucleation sites, T_1_ nucleation and growth could not possibly have taken place without sufficient time and appropriate temperature. This is consistent with work done by the authors of Reference [21], who found high-density dislocations in the as-built sample with no formed T_1_ phases, but reportedly found T_1_ only after T6 heat treatment of the LPBF processed Al-Cu-Li alloy. Through appropriate LPBF preheat treatment (in combination with dissociated dislocation spots which act as nucleation sites), T_1_ phase formation can be achieved through the LPBF process. This puts LPBF in a competitive advantage compared to conventional processing of Al-Cu-Li alloys through the elimination of prior-deformation by cold working with subsequent artificial ageing treatments [20].

Several authors [30,31,32] have also reported that minor alloying elements such as Mg, Zn and Ag are able to facilitate T_1_ nucleation. In work done by the authors of Reference [30], it was indicated that the greatest effect on T_1_ precipitation was by Mg. In all these investigations, the effects of these minor elements were studied with cold working and aging treatment and not in an unconventional manufacturing process like the LPBF. In the current investigations, 0.26 wt.% Mg and 0.67 wt.% Zn could not be said to have facilitated T_1_ nucleation in the LPBF as-built sample, because of the high cooling rate and the resulting lack of appropriate T_1_ formation temperature and time. This further reinforces the point that relying on micro-alloying alone may not be enough to facilitate T_1_ precipitation. However, adopting appropriate T_1_ formation temperature in combination with sufficient nucleation sites arising from dissociated dislocations and/or with key minor elements such as Mg can help facilitate their formation and growth using LPBF processing.

### 3.3. Phase Field Simulation

T_1_ formation temperature in a ternary Al-Cu-Li equilibrium system is reported to be greater than 635 °C [3]. However, in an equilibrium ThermoCalc simulation of an Al-Cu-Li alloy of similar composition to the alloy under investigation [20], T_1_ formation temperature was predicted to approximately range between 240 and 480 °C and that of Scheil-Gulliver between 460 and 520 °C. The authors concluded that LPBF processing using a preheat treatment temperature of 520 °C therefore created favourable conditions for the nucleation and growth of the T_1_ phase, which was suspected to be responsible for improved microhardness over post-heat-treated samples.

**Figure 5:**
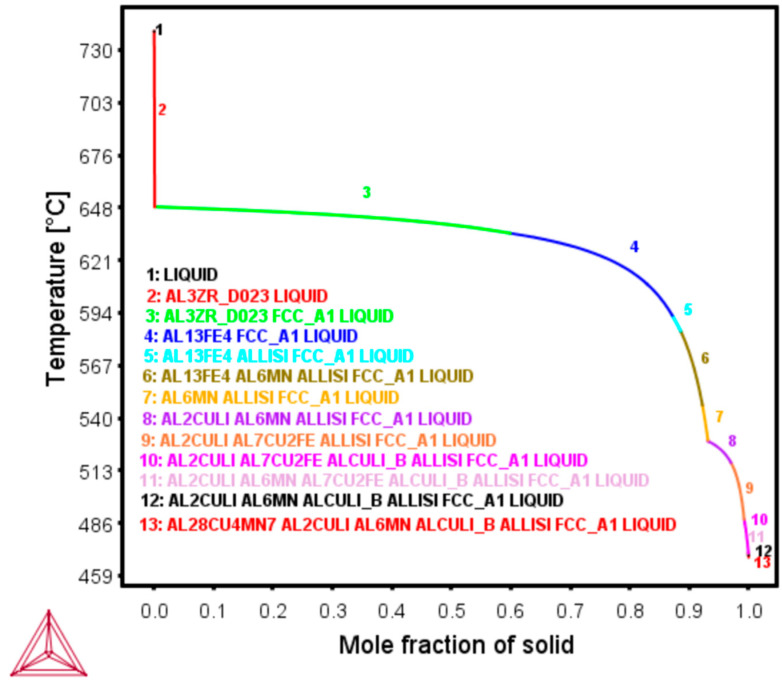
Scheil non-equilibrium simulation (TCal6), predicting T_1_ and T_B_ formation temperatures.

In the Scheil simulation of the alloy in Figure 5 below, T_1_ is similarly expected to start forming around 520 °C, together with other inter-metallics. Subsequently, at about 480 °C, T_B_ is expected to precipitate along with the T_1_ phase down to about 460 °C. Notable phases common to the Al-Li system such as S´, ϴ’ and δ’ are not expected to form based on the Scheil calculations. Subsequent sections will further discuss and correlate the phase field simulations with microstructures and phase characterisations of preheat-treated LPBF (320 and 500 °C) samples.

### 3.4. Microstructure of Preheat-Treated LPBF-Built Sample

**Figure 6:**
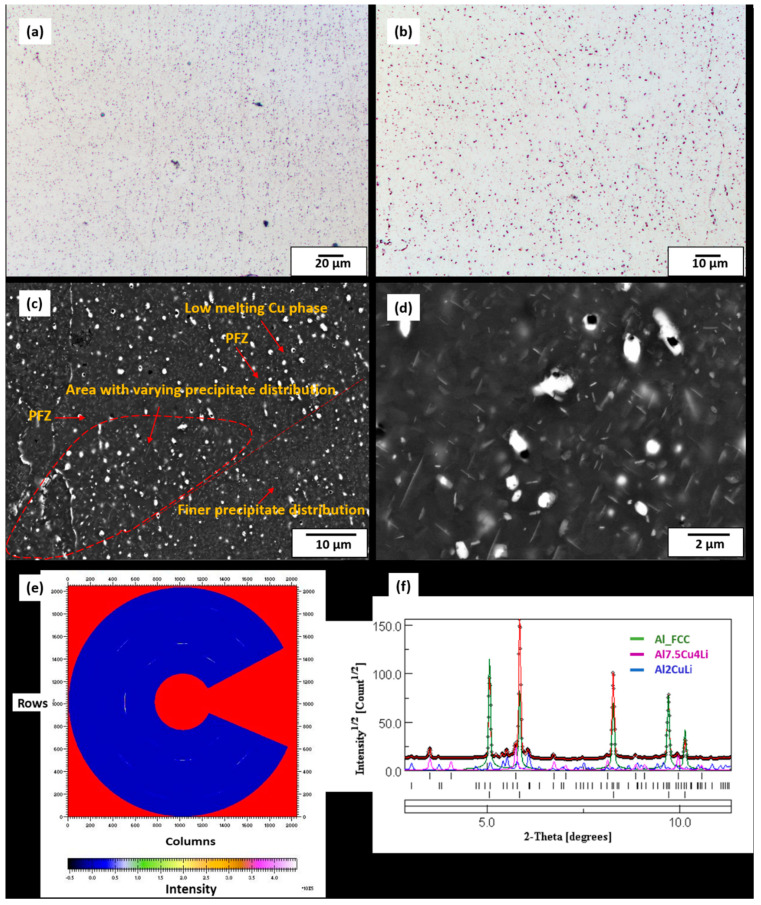
(**a**,**b**) LOM micrographs of etched preheat-treated sample (320 °C). (**c**,**d**) SEM micrographs of preheat-treated sample (320° C) showing rod-like precipitates suspected to be T_1_ and T_B_ plates of varying sizes as well as globular precipitates which appear to be rich in Cu. Synchrotron High-Energy X-ray Diffraction (S-HEXRD) measurements (320 °C sample), (**e**) masked ring pattern, (**f**) diffraction peaks confirming two main dominant phases, T_1_ and T_B_.

LOM microstructures (etched) of 320 °C preheat-treated samples (Figure 6a,b) clearly differ from etched LOM microstructure of the as-built sample (Figure 3a). Contrary to the as-built microstructures, melt-pool boundaries and overlapping melt-pool boundaries are hardly visible after preheat treatment at 320 °C. Heat treatments tend to homogenise microstructures and may have accounted for melt-pool dissolution at 320 °C [33,34]. SEM of 320 °C micrographs shown in Figure 6c,d at different magnification levels show a microstructure with a variety of rod-like precipitates in the matrix, as well as globular-like phases which are seen as bright spots and appear to be Cu-rich. These rod-like precipitates are not only seen to be unevenly distributed, they also vary in size (≤2 µm) and orientation. Since the LPBF process is characterised by localised rapid solidifications [23], disparities in local cooling rates at the preheat treatment temperature (320 °C) may have influenced the varying precipitate morphology across the matrix. Whereas precipitate-free zones (PFZ) are observed in certain portions of the matrix, other portions show sparsely distributed, bigger rod-like precipitates as well as smaller and densely distributed ones (Figure 6d). From the SEM morphological observations, one can suspect these rod-like precipitates to be either T_1_ (Al_2_CuLi) or T_B_ (Al_7.5_Cu_4_Li), which is also confirmed by the S-HEXRD ring pattern and diffraction peaks shown in Figure 6e,f.

The S-HEXRD masked ring patterns (Figure 6e) were first integrated into one-dimensional diffraction peaks, after which the peaks were refined based on the Rietveld refinement method and fitted with their respective phases (Figure 6f). The diffraction peaks from the confirmed T_1_ and T_B_ phases were generally of low intensity. The T_1_ phase formation temperature is predicted to start at elevated temperatures, at about 520 °C according to the Scheil-Gulliver simulation, and continuously down to about 460 °C (Figure 5). Similarly, the Scheil simulation also predicts T_B_ later forming along with the T_1_ phase between 460 °C and 480 °C. At the 320 °C preheat temperature, however, T_1_ and T_B_ rod-like precipitates were still present, since this temperature is in their equilibrium stability range. In conventionally processed alloys, the rod-like T_B_ phase is reportedly formed from the metastable Al_2_Cu (ϴ’) phase by the replacement of Al by Li atoms [35,36]. It was also reported to form during overage treatments at higher temperatures (>350 °C) by the authors of Reference [37] in a conventionally processed AA 2099 alloy and also between 542 and 550 °C for an equilibrium ternary Al-Cu-Li system by the authors of Reference [3]. In the work by the authors of Reference [6], on over-aging and tempered AA 2198, T_B_ was reportedly formed as an equilibrium phase upon the dissolution of phases such as ϴ’ and T_1_. The non-equilibrium LPBF process which was followed by experimental S-HEXRD phase characterisation confirms that the T_B_ phase can be formed under non-equilibrium conditions, and under this investigation, it was found after the preheat treatment temperature of 320 °C.

**Figure 7:**
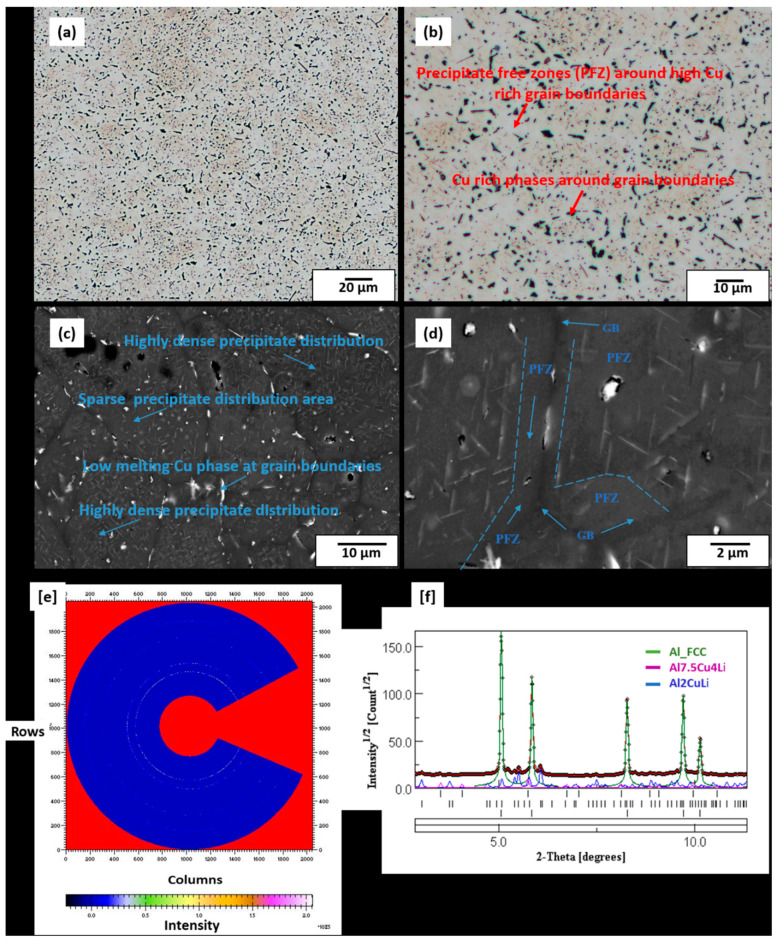
(**a**,**b**) LOM micrographs of preheat-treated sample (500 °C) showing Cu-rich phases at grain boundaries (GB), light colour: Al-rich, dark: Cu-rich, little golden (brown) rod-like precipitates. (**c**,**d**) SEM micrographs of preheat-treated sample (500 °C) showing rod-like precipitates suspected to be T_1_ and T_B_ precipitates of varying sizes and orientations, as well as Cu-rich phases around grain boundaries and Precipitate Free Zone (PFZ). S-HEXRD measurements (500 °C sample), (**e**) masked ring pattern, (**f**) diffraction peaks confirming the two main dominant phases, T_1_ and T_B_.

**Figure 8:**
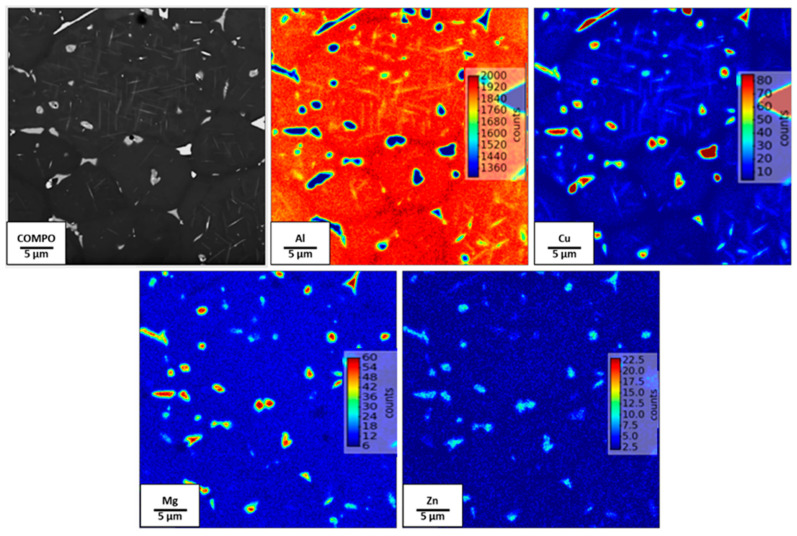
SEM/EPMA maps of preheat-treated sample (500 °C) showing Cu-, Mg- and Zn-enriched phases, and PFZ as well as T_1_ and T_B_ plates.

At the preheat treatment temperature of 500 °C, LOM and SEM micrographs shown in Figure 7a–d highlight the distribution of rod-like precipitates in the matrix. These precipitates, which were similarly found in the 320 °C preheat-treated sample, are confirmed in this sample as T_1_ and T_B_ plates after masking and integrating the two-dimensional ring patterns (Figure 7e) into one-dimensional diffraction peaks (Figure 7f) through S-HEXRD diffraction experiments. The peaks were also refined and fitted according to the Rietveld refinement method, and the identified phases were consistent with the Scheil calculations. PFZ around the grain boundaries as well as grain boundaries of highly rich Cu phases can also be observed. These low-melting Cu-rich phases formed due to the non-equilibrium solidification conditions and the fast cooling rates of the LPBF process, which triggered the movement of vacancy-solute atoms’ complexes towards vacancy sinks, like the grain boundaries and their interfaces [38]. Grain boundary Cu-rich films were also seen in conventionally produced AA2099 Al-Cu-Li alloy and were described as being susceptible for corrosion attack [39]. The EPMA maps in Figure 8 further reveal the segregated Cu-rich phases, which appear as thin films on the grain boundaries of the 500 °C preheat-treated sample. In the 320 °C preheat-treated sample, however, these Cu-phases appeared globular in shape. The Cu-rich phases observed in all the preheat-treated samples also contained significant Mg and Zn concentrations, which is consistent with the EPMA mapping observations of the gas-atomized powder cross-section. Several options can be adopted in the reduction of grain boundary Cu-rich phases as well as the elimination of PFZ. These include grain refinement, alloy chemistry modification, particularly the Cu/Li, Cu/Mg ratio and the precipitation of the S’ (Al_2_CuMg) strengthening phase, which is known to eliminate PFZ in Al-Li alloys [40]. The presence of PFZ around grain boundaries have been widely reported [40,41,42] in conventionally processed Al-Li alloys. They were either attributed to grain boundary misorientation, depleted vacancies around grain boundaries which were conducted towards grain boundary sinks or the depletion of solute around grain boundaries [42]. In this multi-precipitation Al-Cu-Li system, Cu/Li ratio, as well as the presence of other minor solute elements such as Mg, Zr and Ag, among others, can either influence T_1_ precipitation kinetics or its preferential precipitation over other phases [9,43]. Although T_1_ and T_B_ precipitates are also known to form both within the grains and on grain boundaries [9], perhaps the segregation of Cu to grain boundary sinks as shown in Figure 7a–d might have created locally depleted Cu zones around the grain boundaries and deprived those areas of Cu available for the formation of T_1_ and T_B_.

### 3.5. EBSD Characterisation

**Figure 9:**
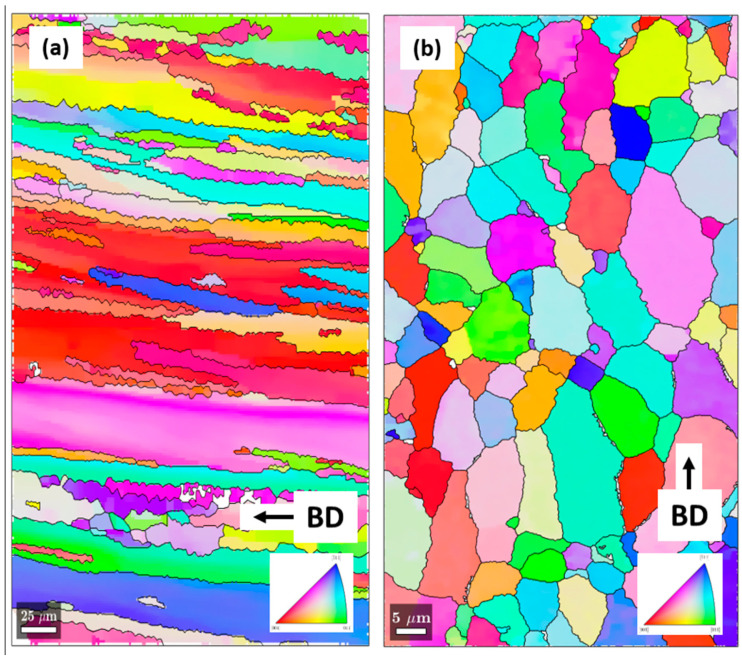
Electron Backscatter Diffraction (EBSD) Inverse Pole Figures (IPF) of: (**a**) 320 °C preheat-treated sample showing elongated grains along the building direction (BD) and (**b**) 500° C preheat-treated sample with a nearly equiaxed grain structure.

Figure 9 highlights and compares EBSD Inverse Pole Figure (IPF) figures of the preheat-treated samples at 320 and 500 °C. A sharp contrast can be observed in terms of grain structure and orientation. It is clearly seen in Figure 9a that the 320 °C preheat-treated sample is characterised by elongated grains which are also oriented along the building direction. The influence of the unidirectional flow of heat flux away from the substrate plate towards the building direction, the temperature gradient thereof and the fast cooling rates which persisted even after preheat treatment of 320 °C can be said to be responsible for the elongated grain growth orientation towards the building direction [44]. Preheat treatment temperature of 320 °C is expected to have slowed down the cooling rate through lowering the temperature gradient (G). The ratio of G to R (rate of solidification), which is predictive of the solidification morphology, was probably not sufficient enough to cause a grain transition into a completely equiaxed structure. A nearly equiaxed grain structure is seen in Figure 9b, of the 500 °C preheat-treated sample. Through the combination of process parameters such as the laser power and scanning speed, coupled with the 500 °C preheat treatment temperature, the cooling rate and the temperature gradient reduced, creating optimum conditions for the growth of nearly equiaxed grains, as observed in the EBSD IPF figure [45].

### 3.6. Microhardness

**Figure 10:**
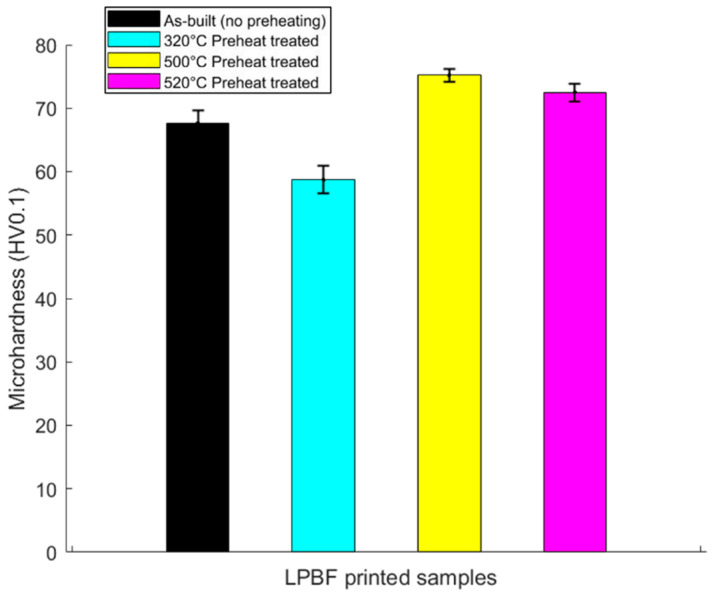
Microhardness of 520 °C preheat-treated sample [20]. Microhardness comparison of as-built, 320 °C and 500° C preheat-treated samples showing elevated microhardness of the 500 °C sample due to higher T_1_ volume fraction, followed by the as-built samples, which is attributed to residual stress build-up.

A comparison of averaged microhardness of as-built, 320 °C and 500 °C preheat-treated samples using a load of 100 g is illustrated in Figure 10 below. The microhardness illustration shows that the highest microhardness was recorded in the 500 °C preheat-treated samples, followed by the as-built samples, and then that of the 320 °C preheat-treated samples. The highest recorded microhardness for the 500 °C preheated samples was 75.2 HV0.1 compared to 58.7 HV0.1 for 320 °C and 67.6 HV0.1 for as-built samples, respectively. The microhardness of 72 HV0.1 for 520 °C preheat-treated samples in our earlier work [20], shows that it is possible to achieve a relatively higher microhardness with a lower preheat treatment temperature (500 °C). The build-up of residual stresses in the LPBF-built part, typically in the as-built condition, is due to the high-temperature gradient, thermal contraction and expansion or the lack of uniformity in plastic deformation as a result of heating and cooling cycles, and is known to boost hardness properties [46]. High residual stresses can equally be detrimental to part properties, inducing residual stress-related cracks, as shown earlier in this work. As stated earlier, stress relief, preheat treatment or the adoption of appropriate scanning strategies are possible mitigation steps which can be adopted to deal with the detrimental effects of residual stresses. Since no known strengthening phases were detected in the characterised as-built microstructure, the relatively high microhardness (67.6 HV0.1) can be attributed to the build-up of large amounts of residual stress.

From our earlier work, post heat treatment rather than pre-heat treatment on the precipitation hardenable alloy [20] proved to have detrimental effects on the mechanical properties (microhardness). In this work, however, for both preheat-treated samples (320 °C, 500 °C), the 500 °C samples recorded superior relative density and microhardness. Quantitative compositional phase measurements by MAUD software shows that while the volume fraction of the T_B_ phase was higher at 320 °C than at 500 °C preheat treatment temperature, the T_1_ phase was conversely slightly higher in volume fraction at 500 °C preheat treatment temperature. A T_B_ volume fraction of 4.2% was recorded at 320 °C and 0.34% at 500 °C, while T_1_ volume fraction was 2.1% at 320 °C and 2.9% at 500 °C preheat treatment temperatures, respectively. The contribution of T_B_ phase to the strength of Al-Cu-Li alloys is regarded as not significant [6] and that explains why although a higher volume fraction was recorded in the 320 °C samples, it possessed a lower microhardness, as seen in Figure 10. The other possible reason is process-related defects from pores and cracks, which were also observed, although this is considered as having minimal contributing effect. The higher microhardness measured at the 500 °C preheat treatment is attributed to the comparatively finer grain structure shown by the EBSD IPF figure, the higher part relative density, and, most significantly, the strengthening effect of the T_1_ phase, which has been confirmed by various authors [5,12,47,48]. This is also evidenced by the slightly higher T_1_ volume fraction recorded at the 500 °C preheat treatment temperature and confirms our earlier work [20] on AM (LPBF)-processed AA2099 Al-Cu-Li alloy, where increased microhardness was attributed to the strengthening effect of the T_1_ phase after 520 °C preheat treatment temperature. The authors of Reference [21], after LPBF processing of AA2195 and performing a T6 thermal treatment, also attributed improvement in mechanical properties to the T_1_ phase. The T_B_ phase is, however, in this work, reported for the first time in an AM (LPBF)-processed Al-Cu-Li alloy, although its strengthening effect is regarded as not significant. The precipitation of a high-volume fraction of strengthening phases, including T_1_, is therefore consequential to improving the mechanical properties of the alloy.

## 4. Conclusions

The Al-Cu-Li alloy is a multi-precipitation system with a precipitation kinetics dependant on several factors: (a) alloy chemistry (Cu:Li ratio, Cu:Mg ratio and the presence of Mn, Zr, Zn or Ag) and (b) appropriate heat treatment strategies that help facilitate the formation of several phases, including the main strengthening phase, T_1_ (Al_2_CuLi). The T_1_ formation through conventional manufacturing methods using various forms of thermomechanical treatments requires prior deformation by cold working with appropriate aging treatments. There has been extensive reporting on the microstructure of conventionally processed Al-Cu-Li alloys but not much is known of their AM-characterised microstructure. Considering the enormous lightweight potential of the Al-Cu-Li alloy system, the alloys’ strength and properties can be maximized for AM applications through a clear correlation between the micro- and macro-structure properties. This can be achieved first of all through a gainful understanding of their AM microstructure resulting from process parameters and a careful tailoring of the microstructure through the precipitation of a high-volume fraction strength contributing phase.

This work utilised a combination of various analytical measurements including S-HEXRD to characterise the microstructure of as-built (no preheating) and preheat-treated AA2099 Al-Cu-Li LPBF-built samples. No post-heat treatment was performed.

In the as-built microstructure, cracks were observed (Figure 3), which are attributed to residual stress build-up. TEM investigations showed dislocations with no detection of the T_1_ phase, consistent with observations in Reference [21]. This confirms the conventional view that T_1_, even at dislocation sites, still finds it difficult to nucleate without appropriate time and heat treatment temperatures.

In the 500 °C preheat-treated samples, Cu-rich phases which appeared as films on the grain boundaries were surrounded by PFZ. The Cu-rich phases caused Cu-depleted areas around the grain boundaries. In the 320 °C preheat-treated sample, Cu-rich phases appeared as globular phases. The PFZ around the low-melting Cu-rich phases at the grain boundaries are attributed to the movement of vacancy and Cu solute atoms to grain boundary sinks during solidification. Cu-depleted areas (PFZ) around the grain boundaries inadvertently affect the local Cu:Li and Cu:Mg ratios, which are critical for T_1_ phase formation. An interplay between the Cu:Mg as well as Cu:Li ratios can facilitate the precipitation of grain boundary precipitates such as S and T_1_ phase and minimize the PFZ effect around the grain boundary. Minimizing grain boundary Cu-rich phases as well as PFZ around grain boundaries is a main goal, because they lead to susceptible corrosion attack [39] and also reduce the fracture toughness of the alloy [41].

Through preheat treatment temperatures at 320 and 500 °C, two main dominant phases (T_1_–Al_2_CuLi and T_B_–Al_7.5_Cu_4_Li) were detected with S-HEXRD homogenously distributed at different volume fractions and sizes. Because T_B_ phase (Al_7.5_Cu_4_Li) is a stabilised form of metastable Al_2_Cu (ϴ’) and known to form at its expense [16], it was not surprising that no ϴ’ phase was detected with the S-HEXRD. Microhardness was comparatively higher in the 500 °C preheat-treated samples than in the 320 °C preheat-treated samples. This is attributed to the higher volume fraction of the T_1_ phase (2.9%) in the 500 °C preheat-treated sample and not the strengthening effect of the T_B_ phase, which is considered as minimal. Although a higher volume fraction of the T_B_ phase (4.2%) was measured in the 320 °C preheat-treated samples than in the 500 °C samples, a lower microhardness was recorded (Figure 9). Scheil simulation performed for this alloy composition indicated that T_1_ phase starts first at a higher temperature, and T_B_ phase follows afterwards, concurrently with the T_1_ phase. It is suggestive to conclude that if the T_B_ phase is a stabilised form of the Al_2_Cu (ϴ’) phase, forming at its expense [49], and subsequently forming concurrently with the T_1_ phase, then it deprives the alloy of strengthening phases such as the metastable Al_2_Cu (ϴ’) phase and the main strengthening T_1_ phase. The stabilisation of the Al_2_Cu (ϴ’) phase into T_B_ is reported to take place by the replacement of Al atoms by Li atoms [50], and it is therefore plausible to conclude that local Li atoms which should be available for the precipitation of a high-volume fraction of T_1_ could be lost to the T_B_ phase since they are both Li-bearing phases.

Therefore, future work will investigate T_B_ suppression at the expense of strength-contributing phases such as ϴ’ and T_1_ to optimise the strength and ductility properties of the alloy. Through modification of the alloy chemistry and appropriate heat treatment strategies, a high-volume fraction of finely distributed T_1_ precipitates can be formed at the expense of other minimal strengthening contributing phases like T_B_. Further investigations are therefore recommended in the finding of optimum conditions for forming fine and homogenously distributed T_1_ precipitates which are critical to achieving peak strength through AM processing.

Grain refinement proved to be effective in the removal of cracks in the LPBF-built AA2099 alloy [20]. This provides a pathway for further investigations for lowering preheat treatment temperatures and inhibiting cracks, as well as achieving the precipitation of strengthening phases through modification of the alloy chemistry.

The next phase of this work will also focus on the deployment of other analytical instruments, including Atom Probe Tomography (ATP) and Scanning Transmission Electron Microscope (STEM), to conduct a local precipitate investigation/characterisation, especially of T_1_, as found in the LPBF-built sample.

## Figures and Tables

**Table 1:** Nominal composition (wt.%) of gas-atomized AlCu2.7Li1.8Mg0.3 powder.

Material	Nominal Composition (wt.%)							
AlCu2.7Li1.8Mg0.3	Al	Cu	Li	Zn	Mg	Mn	Zr	Si
-	Bal	2.63	1.56	0.67	0.28	0.17	0.09	0.02

**Table 2:** Laser Powder Bed Fusion (LPBF) process parameters of densest test samples.

Parameter Table for Printed LPBF Samples					
LPBF Built Condition	Laser Power (W)	Scanning Speed (mm/s)	Hatch Distance (µm)	Layer Thickness (µm)	Relative Density (%)
As-built sample (no preheating)	200	650	160	30	99.0
Preheat-treated sample, 320 °C	170	500	140	30	98.5
500 °C	120	500	140	30	99.6
